# Automation and integration of a novel restricted single‐isocenter stereotactic body radiotherapy (*a*‐RESIST) method for synchronous two lung lesions

**DOI:** 10.1002/acm2.13259

**Published:** 2021-05-25

**Authors:** Lana Sanford Critchfield, Justin Visak, Mark E Bernard, Marcus E Randall, Ronald C McGarry, Damodar Pokhrel

**Affiliations:** ^1^ Medical Physics Graduate Program Department of Radiation Medicine University of Kentucky Lexington KY 40508 USA

**Keywords:** automation, lung SBRT, RESIST, single‐isocenter VMAT, synchronous multiple lesions

## Abstract

Synchronous treatment of two lung lesions using a single‐isocenter volumetric modulated arc therapy (VMAT) stereotactic body radiation therapy (SBRT) plan can decrease treatment time and reduce the impact of intrafraction motion. However, alignment of both lesions on a single cone beam CT (CBCT) can prove difficult and may lead to setup errors and unacceptable target coverage loss. A Restricted Single‐Isocenter Stereotactic Body Radiotherapy (RESIST) method was created to minimize setup uncertainties and provide treatment delivery flexibility. RESIST utilizes a single‐isocenter placed at patient’s midline and allows both lesions to be planned separately but treated in the same session. Herein is described a process of automation of this novel RESIST method. Automation of RESIST significantly reduced treatment planning time while maintaining the benefits of RESIST. To demonstrate feasibility, ten patients with two lung lesions previously treated with a single‐isocenter clinical VMAT plan were replanned manually with RESIST (*m‐*RESIST) and with automated RESIST (*a*‐RESIST). *a*‐RESIST method automatically sets isocenter, creates beam geometry, chooses appropriate dose calculation algorithms, and performs VMAT optimization using an in‐house trained knowledge‐based planning model for lung SBRT. Both *m*‐RESIST and *a*‐RESIST showed lower dose to normal tissues compared to manually planned clinical VMAT although *a*‐RESIST provided slightly inferior, but still clinically acceptable, dose conformity and gradient indices. However, *a*‐RESIST significantly reduced the treatment planning time to less than 20 min and provided a higher dose to the lung tumors. The *a*‐RESIST method provides guidance for inexperienced planners by standardizing beam geometry and plan optimization using DVH estimates. It produces clinically acceptable two lesions VMAT lung SBRT plans efficiently. We have further validated *a*‐RESIST on phantom measurement and independent pretreatment dose verification of another four selected 2‐lesions lung SBRT patients and implemented clinically. Further development of *a*‐RESIST for more than two lung lesions and refining this approach for extracranial oligometastastic abdominal/pelvic SBRT, including development of automated simulated collision detection algorithm, merits future investigation.

## 1 INTRODUCTION

Stereotactic body radiation therapy (SBRT) of synchronous multiple primary or metastatic lung lesions can result in excessively long treatment planning and delivery times for patients and busy clinics. To alleviate this process, a single‐isocenter intensity modulated radiation therapy (IMRT) or volumetric modulated arc therapy (VMAT) SBRT plan is a feasible treatment option for patients presenting with synchronous multiple metastatic or primary lung lesions.^1^, ^2^, ^3^, ^4^, ^5^ SBRT of two lung lesions with a single‐isocenter VMAT plan significantly decreases treatment delivery time, increases patient comfort and compliance, and reduces the chance of intrafraction tumor motion errors.^5^ However, small patient setup errors may occur due to the difficulties of multiple lung lesions alignment on a single cone beam CT (CBCT) scan. These small setup errors may lead to unacceptable loss in target(s) coverage due to lung heterogeneities and the steep dose gradients obtained in the SBRT plan.^6^ Thus, a Restricted Single‐Isocenter Stereotactic Body Radiotherapy (RESIST) method was developed to minimize the problems associated with a single‐isocenter VMAT lung SBRT plan (e.g., setup errors, collision issues).^7^ It has been reported on a multi‐institutional database of 700 patients treated with SBRT that patient outcome is related to a clinic’s experience in delivery of SBRT.^8^ There are no definitive treatment planning guidelines for inexperienced clinics in the treatment of multiple lesions lung SBRT who wish to treat their patients efficiently and accurately.

Recently, a few investigators have presented their work on the use of automation for generating lung SBRT treatment plans using a knowledge‐based planning (KBP) approach with dose volume histogram (DVH) estimates via RapidPlan (RP) modeling (Varian Medical Systems, Palo Alto CA).^9^, ^10^, ^11^ KBP models can generate plans quickly and improve plan quality and consistency by reducing interplanner variability. These models are trained using previously treated high quality treatment plans and provide a good starting point for subsequent plan optimization. However, there has yet to be a KBP model to automate treatment planning for multilesion lung SBRT including isocenter placement, deploying beam geometry, assigning appropriate dose calculation algorithm and optimizing the plan. In order to guide planners in generating single‐isocenter/multi‐lesions VMAT lung SBRT plans, an automated treatment planning routine (*a*‐RESIST) has been developed using the RESIST planning geometry, which is further optimized using an in‐house trained KBP lung SBRT model.^11^ In the *a*‐RESIST plan, placement of single‐isocenter at the mediastinum avoids potential patient/gantry collisions, provides greater flexibility of noncoplanar partial arcs geometry and eliminates the need for multiple couch movements during CBCT imaging. In between the plans, the therapists do not need to enter the treatment vault to reposition the patient because the *a*‐RESIST plans share the same isocenter and the isocenter placement at patient midline ensuring that the daily CBCT imaging will clear the patient without applying a couch shift (couch shift needed for Varian Linac for off‐center patients >5 cm laterally). Thus, *a*‐RESIST reduces the chance of a geometric miss due by allowing daily pretreatment CBCT soft tissue matching of one tumor at a time. Moreover, the physician can choose to treat only one lesion per treatment without causing any error in dose tracking (if needed) to manage the patient for various clinical reasons, such as reducing the lung toxicity or if patient cannot tolerate the entire course of treatment. This report aims to demonstrate the feasibility of the *a‐*RESIST treatment planning technique and its ability to assist planners in improving planning efficiency, consistency, and accuracy. Furthermore, this report also provides guidance for automating treatments and simplified workflow for the therapists for the fast and effective synchronous multiple lesions lung SBRT–potentially allowing for offline adaptive replanning, if required.

## 2 MATERIALS AND METHODS

### 2.A Phantom measurements

First, the independent dose validation was performed using the MD Anderson’s SBRT credentialing phantom with two targets (spine and lung) by delivering a SBRT prescription dose of 6.0 Gy to the both targets using a single‐isocenter VMAT plan following NRG‐BR001 protocol.^1^ Distance between the spine and lung targets were about 9 cm apart. All dosimetric criteria established by IROC for SBRT treatments to multilesions using single‐isocenter approach were satisfied. Second, for our TrueBeam Linac, to quantify the spatial positioning accuracy of a single‐isocenter/multitargets plan as a function of distance from the isocenter, the end‐to‐end phantom tests were performed. Because of the lack of specialized multitargets phantom in our center, for the end‐to‐end tests, we utilized clinically available catphan phantom with multiple imaging inserts at the different planes and the MPC phantom with 16 bearing balls (BBs) at the different locations. On these phantoms’ measurements, it has been observed that our CBCT based target localization accuracy on our Truebeam Linac was within 1 mm (average, 0.75 mm) at 7 cm and <1.2 mm (average, 0.81 mm) at 10 cm distance from the isocenter respectively.

### 2.B Patient CT simulation and contouring

After obtaining institutional review board approval, patients were retrospectively selected with two lung tumors each who were previously treated to 50 Gy in 5 fractions using a single‐isocenter VMAT lung SBRT following RTOG guidelines.^12^ For each patient, both lesions were treated at the same time every other day. All patients were immobilized with the Body Pro‐Lok^TM^ SBRT system (CIVCO, Orange City, IA) in the supine position with arms above their head. A simulation CT scan was obtained on a GE Lightspeed 16 slice CT scanner (General Electric Medical Systems, Waukesha, WI) with 512 × 512‐pixel image size and 1.25 mm slice thickness in the axial helical mode. For respiratory motion control, most patients tolerated abdominal compression, if not a 4D‐CT scan was obtained by utilizing Varian RPM system (version 1.7). Images were imported into the Eclipse Treatment Planning System (TPS, Version 15.6, Varian Medical Systems, Palo Alto, CA) for contouring.^13^ Gross tumor volumes (GTVs) were delineated based on the observable tumor mass. If a 4D‐CT was obtained, internal target volumes (ITVs) were contoured based on maximum intensity projection (MIP) co‐registered with the free breathing planning CT images in Eclipse TPS. A planning target volume (PTV) was created by expanding the GTVs by 10 mm in the superior‐inferior direction and 5 mm in the lateral directions (abdominal compression) or expanding the ITVs with a uniform 5 mm margin (4D‐CT). Critical structures were contoured on free‐breathing CT, including normal lung (right, left, and combined), spinal cord, heart, bronchus, trachea, esophagus, skin, and individual ribs (right, left, and combined) per RTOG requirements.^1^, ^12^, ^14^ Table 1 summarizes the tumor characteristics and tumor distance to isocenter for the ten multilesions lung SBRT patients included in this study. Four lesions were within 2 cm distance from the principal bronchial tree. Distance to isocenter was calculated by finding the Cartesian coordinates of the each PTV geometric center and determining the Euclidian 3D distance with the isocenter coordinates for each plan. Due to the limited field‐of‐view (2–3 cm superior to inferior direction for the tumor location) of our 4D‐CT scan, our clinical treatment plans were generated on the free breathing planning CT images.

**Table 1:** Main tumor characteristics of the ten lung SBRT patients included in this study. Each patient had two tumors. STD = standard deviation.

Parameters and plans		Mean ± STD (range or n = no. of patients)
Tumor 1,	GTV1 (cc)	5.3 ± 6.6 (0.6 – 24.6)
	PTV1 (cc)	21.4 ± 17.2 (6.5 – 69.6)
Tumor 2,	GTV2 (cc)	5.5 ± 5.0 (0.6 – 15.8)
	PTV2 (cc)	22.0 ± 13.1 (6.4 – 40.9)
Prescribed dose to each lesion		50 Gy in 5 fractions
Tumor location (left/right/bi‐lateral lungs)		(n = 4 / 1 / 5)
Tumors located < 2 cm from the bronchial tree		4
Normal lung (cc)		3837.3 ± 1171.2 (2041 – 6542)
Isocenter to tumor distance (cm)	Clinical plans	5.5 ± 2.3 (2.4 – 9.2)
	*m‐*RESIST plans	7.4 ± 2.0 (3.2 – 11.3)
	*a‐*RESIST plans	8.1 ± 2.1 (4.5 – 10.9)

### 2.C Clinical VMAT plans

All these patients were treated using a clinical single‐isocenter lung SBRT plan that was generated in Eclipse TPS using a Truebeam Linac (Varian Medical Systems, Palo Alto, CA) with the Millennium 120 MLC. All VMAT plans were generated manually utilizing 6 MV‐FFF (1400 MU/min) beams. The isocenter was placed approximately between the two tumors. For patients who presented with bilateral tumors or select unilateral tumors, two to three full co‐planner arcs were used for treatment. For the remaining unilateral cases, three to five partial co‐planner or noncoplanar arcs with couch rotations up to ± 10° were utilized (planner preference). Collimator angles were manually chosen to reduce the MLC leakage dose between each arc with the jaw‐tracking feature enabled.^15^ Dose was 50 Gy in 5 fractions for all patients. Target naming convention (PTV1 or PTV2) was chosen by the treating physician. Both PTVs were planned with dose prescribed to the 70–80% isodose lines and optimized such that at least 95% of each PTV received 100% of the prescription dose. The maximum dose to each target was planned to fall inside the GTV. Dose was calculated using the Boltzmann transport based AcurosXB algorithm in Eclipse with heterogeneity corrections with a 1.25 mm calculation grid size (CGS).^13^ Reporting dose to medium and photon optimizer (PO) MLC algorithm was used. Although single‐isocenter SBRT was designed for synchronous treatment of two lesions, planning objectives per RTOG protocols and NRG‐BR001 were utilized for the organs‐at‐risk (OAR).^1^, ^12^, ^14^ Each patient was treated every other day with the VMAT planning technique using an in‐house CBCT‐guided lung SBRT protocol.

### 2.D *m‐*RESIST VMAT plans

Each patient’s clinical treatment plan was replanned using the manual RESIST (*m*‐RESIST) method. The *m*‐RESIST method places isocenter at the patient’s midline and both tumors share the treatment isocenter. If the lesions are separated in the x‐direction, the isocenter is placed approximately between the lesions in the mid‐coronal plane of the patient. A separate plan is made for PTV1 and PTV2. Each plan has three partial noncoplanar VMAT arcs with a 6 MV FFF (1400 MU/min) beam deployed on the tumor side of the patient. Couch rotations were 0°, 10°, and 350° for each beam respectively. Collimator angles were offset by 30° to reduce leakage dose in the same plane and were chosen to ensure that the MLCs can travel to the PTV locations. The new aperture shape controller (ACS) feature in the PO MLC algorithm was set to “very high” in order to reduce the total number of monitor units, reduce plan complexity, and improve plan quality as demonstrated by the previous researchers.^16^, ^17^ Briefly, m‐RESIST plans were created by fitting the MLCs to PTV1 and then calculating the dynamic conformal arc (DCA) dose for the respective plan. Next, standard manual VMAT optimization began for PTV1 and GTV1 coverage. The jaw tracking option was employed to reduce leakage dose to normal lung as described above.^16^ Once dose calculation was complete, the plan for PTV1 was used as a base‐dose plan before VMAT optimization in the plan for PTV2. The PTV2 plan was optimized for coverage to PTV2 and GTV2 and to spare the OAR. Once optimized and calculated, a *m*‐RESIST plan summation was created with both plans and re‐normalized to account for contribution from each plan. The plans were then evaluated per lung SBRT protocols.

### 2.E *a‐*RESIST VMAT Plans

Varian Eclipse Scripting Application Programming Interface (ESAPI, Version 15.5) allows for integration of writable scripts and supports the automation of SBRT plans.^18^ A script (*a*‐RESIST) was developed using Microsoft Visual Studio written in C# with guidance from the Varian APIs handbook.^19^ Running the script in external beam planning will begin the *a*‐RESIST treatment planning method and selects the appropriate patient’s planning CT dataset and structure set. Utilizing *a*‐RESIST automation requires precise structure naming convention chosen based on institution standards (such as PTV1, PTV2, Lt ribs etc.). The *a*‐RESIST automation routine includes the following:

1. Creation of treatment course *AutoPlan RESIST* and creation of two plans: *RESIST PTV1* and *RESIST PTV2*.
2. Placement of single treatment isocenter with x‐ and y‐ based on the coordinates of the spinal cord contour and z‐ being the axial plane between the two PTVs.
3. Selection of machine (Truebeam Linac), energy (6MV FFF), VMAT arc geometry, and dose rate (1400 MU/min).
4. Creation of three partial arcs from 0° to 180° arc length (CW and CCW direction) on the PTV side of the patient with 0° and ±10° couch positions.
5. Offset collimator angles based on PTVs distance to isocenter to allow for optimal MLC travel distance to PTVs.
6. Selection of appropriate dose calculation algorithms (AcurosXB, PO, VMAT optimization, DVH Estimates algorithm).
7. Application of normal tissue objective and jaw tracking to be used in optimization.
8. Optimization of plans using an in‐house KBP model for lung SBRT.^11^

**Fig. 1:**
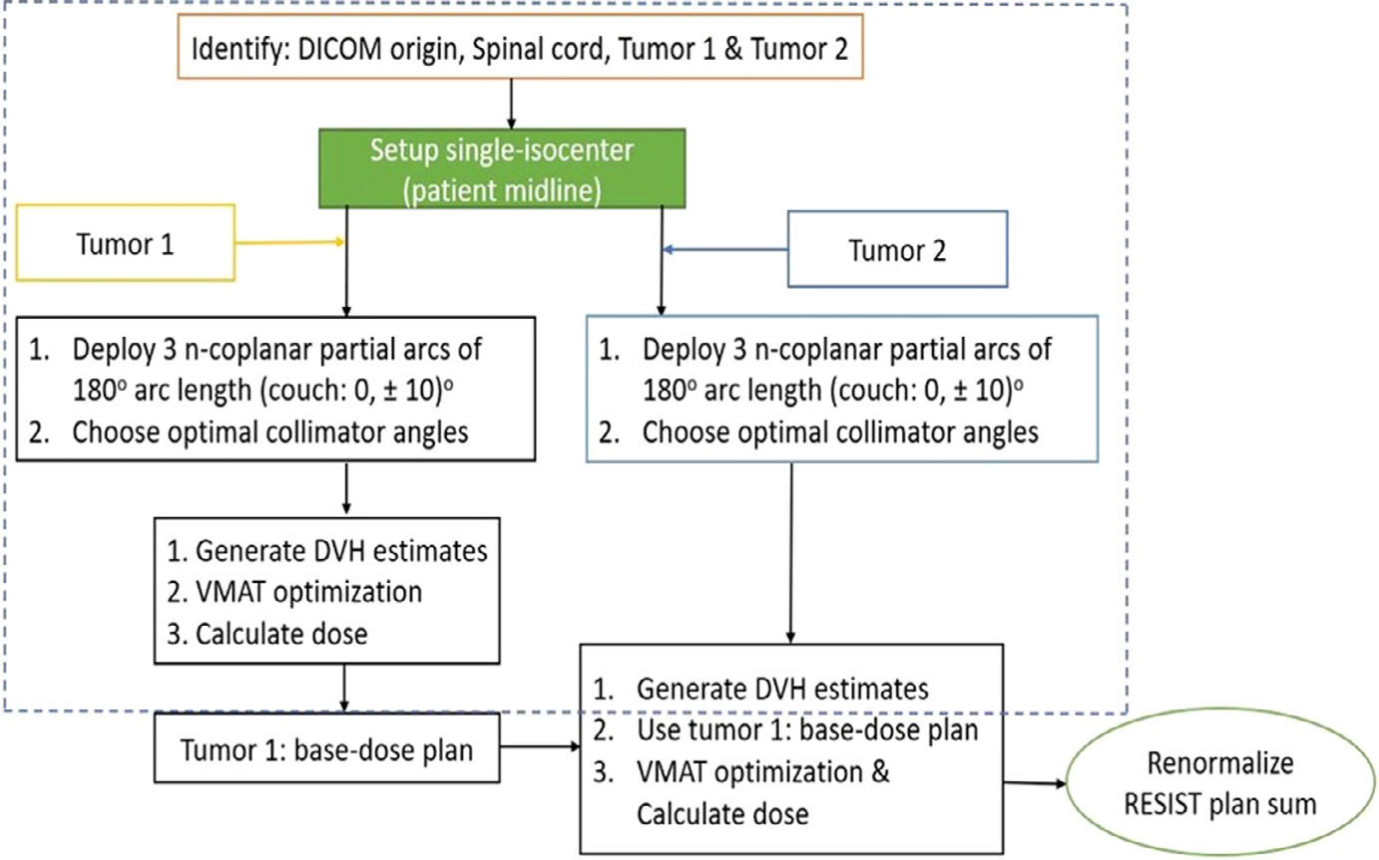
The *a‐*RESIST treatment planning workflow for a single‐isocenter/two lesions VMAT lung SBRT. The selections in the dashed blue box were deployed by the automated treatment planning script with DVH estimates. Utilizing *a‐*RESIST reduces the treatment planning time (< 20 min) significantly and ensures standardized plans for synchronous multilesion lung SBRT.

Briefly, the RTOG‐0813 compliant in‐house KBP lung SBRT model was trained using 86 clinically treated high‐quality noncoplanar VMAT plans for a prescription dose of 50 or 55 Gy in 5 fractions. This model was verified independently and further validated by using another 20 clinical noncoplanar VMAT plans and released for clinical use in our center.^11^ Figure 1 demonstrates the automated (dashed box) and user input sections of the planning workflow for *a*‐RESIST by incorporating the KBP model by generating the DVH estimates for each lesion. After dose calculation of the first plan (*RESIST PTV1*), this plan is chosen as the base‐dose plan for the second plan (*RESIST PTV2)*. The second plan is optimized and dose is calculated. A plan summation is then created by the user and plans are renormalized (if needed) together such that at least 95% of each PTV receives 100% of the prescription dose. The user can adjust the plans from there, further reoptimizing the plans (if needed) including adjusting the beam geometry for better coverage or normal tissue sparing.

### 2.F Plan comparison and data analysis

Plans were compared per RTOG guidelines for conformity index (CI), the ratio of prescription isodose volume to the PTV volume, gradient index (GI) the ratio of the 50% isodose volume to the PTV volume and intermediate dose spillage at 2 cm away in any direction for each target (D2cm).^1^, ^12^, ^14^ Additionally, Paddick conformation number (PCN) was calculated for each target using the Paddick’s formula.^20^ To assess the hot spots of the each plan, heterogeneity index (HI) was calculated as the ratio of PTV maximal dose in cGy and prescription dose. Minimum, maximum, and mean dose to each GTV was assessed. The modulation factor (MF) was defined as the total number of MU divided by the prescription dose in cGy. Doses to OAR that were evaluated included maximum dose to 0.03 cc of ribs, spinal cord, heart, bronchial tree, esophagus, and skin. Volumetric doses to OAR’s were also evaluated for 1 cc of ribs, 0.35 cc of spinal cord, 15 cc of heart, 4 cc of bronchial tree/trachea, 5 cc of esophagus, and 10 cc of skin. Lung doses were assessed for percentage of normal lung receiving 10 Gy (V10 Gy) and 20 Gy (V20 Gy) or more and the mean lung dose (MLD). Moreover, overall treatment planning time was estimated for all three plans. Statistical analysis was performed using Microsoft Excel (Microsoft Corp, Redmond WA) program. Mean, standard deviation (STD), and range values for each of the dose metrics were compared for all three SBRT plans.

## 3 RESULTS

### 3.A Target coverage and dose to OAR

All *a*‐RESIST plans demonstrated acceptable target coverage per SBRT protocols, as shown in Table 2. For similar target coverage, *a‐*RESIST plans provided slightly inferior CI and D2cm compared to both clinical VMAT and *m‐*RESIST plans; however, GI was slightly better with *a‐*RESIST plan. The large GI values reported for clinical VMAT are due to dose bridging between lesions that is eliminated using RESIST methods. Likewise, *a‐*RESIST show slightly inferior PCN compared to *m‐*RESIST plan. These discrepancies are likely attributed to the use of newly created KBP model generating *a‐*RESIST plans slightly inferior to manually optimized treatment plans. This slight degradation can be accounted for given that the KBP model was originally developed for a single‐lesion SBRT plans, i.e. the input data in the model did not include plans that included a base dose thus slightly affecting the model performance. However, the KBP model helped to produce *a‐*RESIST plans with higher GTV minimum, maximum, and mean doses compared to the other planning strategies (see Table 2). This dose escalation is desirable in SBRT treatment since the normal tissue dosing was still acceptable. For instance, the mean GTV dose for *a‐*RESIST was 7% and 6% higher (up to 3.5 Gy) compared to *m‐*RESIST and clinical VMAT plans respectively.

**Table 2:** Analysis of the target coverage of the dosimetric parameters for 10 lung SBRT patients. Mean ± STD (range). STD = standard deviation. PCN = Paddick Conformation Number. CI = conformity index. HI = heterogeneity index. n = number of targets.

Target	Parameter	Clinical VMAT	*m‐*RESIST	*a‐*RESIST
PTV (n = 20)	% Vol. covered by Rx dose (%)	96.2 ± 1.0 (94.8–99.0)	95.8 ± 0.4 (95.1–96.8)	95.9 ± 0.7 (95.2–98.1)
	CI	1.04 ± 0.05 (0.97–1.21)	1.02 ± 0.03 (0.97–1.09)	1.06 ± 0.03 (0.99–1.16)
	PCN	0.88 ± 0.03 (0.81–0.94)	0.90 ± 0.02 (0.85–0.93)	0.88 ± 0.02 (0.80–0.92)
	HI	1.21 ± 0.03 (1.15–1.26)	1.19 ± 0.04 (1.16–1.28)	1.26 ± 0.04 (1.20–1.34)
	GI	6.13 ± 3.20 (3.60–17.6)	4.83 ± 0.76 (3.53–6.28)	4.56 ± 0.65 (3.71–5.93)
	D2cm (%)	54.4 ± 5.8 (47.6–67.0)	51.0 ± 3.7 (44.9–61.3)	55.2 ± 4.9 (43.7–64.3)
GTV (n = 20)	Minimum dose (%)	107.9 ± 3.5 (102.8–114.4)	106.5 ± 4.5 (98.9–115.5)	114.6 ± 3.3 (106.8–120.9)
	Maximum dose (%)	121.1 ± 2.9 (114.8–125.5)	118.8 ± 3.9 (113.4–128.1)	126.1 ± 4.2 (120–134.1)
	Mean dose (%)	114.3 ± 2.1 (111.5–119.2)	113.0 ± 3.6 (107.8–122.6)	120.4 ± 4.9 (106.9–130.3)

**Fig. 2:**
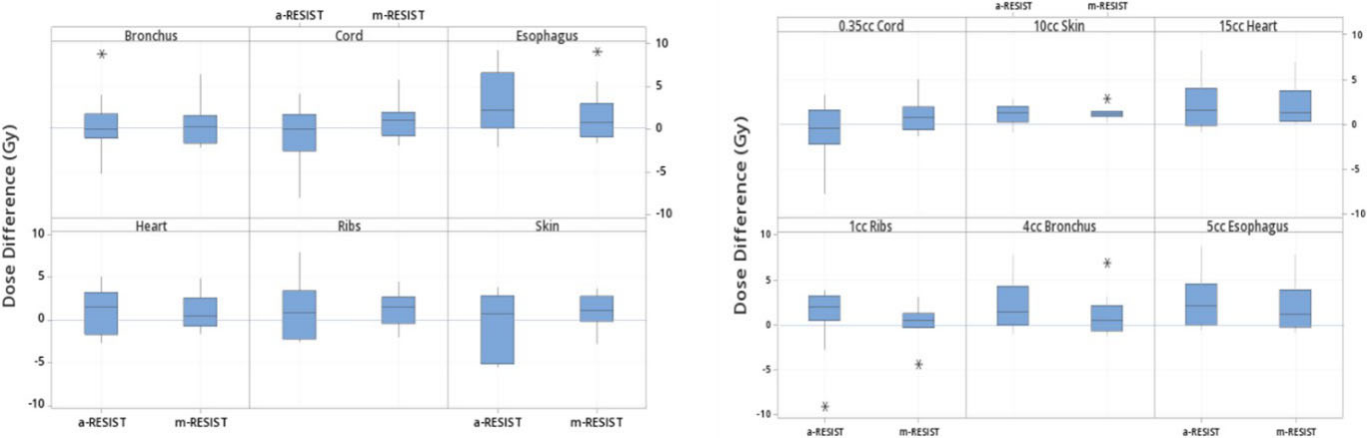
The pairwise differences of *a‐*RESIST and *m*‐RESIST plans with respect to the previously treated single‐isocenter clinical VMAT plans for maximum (left panel) and volumetric (right panel) doses to the OAR. The stars represent outlier data points. *a‐*RESIST provided similar dose to the OAR including sparing principle bronchus tree up to maximal dose of 2 Gy in some cases.

Figure 2 demonstrates the pairwise differences of the dose to OAR for *m‐*RESIST and *a*‐RESIST plans with respect to the original clinical single‐isocenter VMAT plans. The average difference between clinical VMAT and *m*‐RESIST for all maximum and volumetric OAR doses is a positive value, suggesting that in all cases the doses to OAR were lower using *m*‐RESIST compared to clinical VMAT plans. In comparison, the majority of the average differences between clinical VMAT and *a*‐RESIST were positive values, including reducing maximal bronchial tree dose of up to 2 Gy in some cases. However, the average difference for 0.03 and 0.35 cc of spinal cord and 5 cc of esophagus were all negative values, at −0.7, −0.8, and −0.4 Gy respectively. These small OAR sparing discrepancies (<1.0 Gy) suggest that *a*‐RESIST plans were as good as clinical VMAT and *m*‐RESIST plans.

**Fig. 3:**
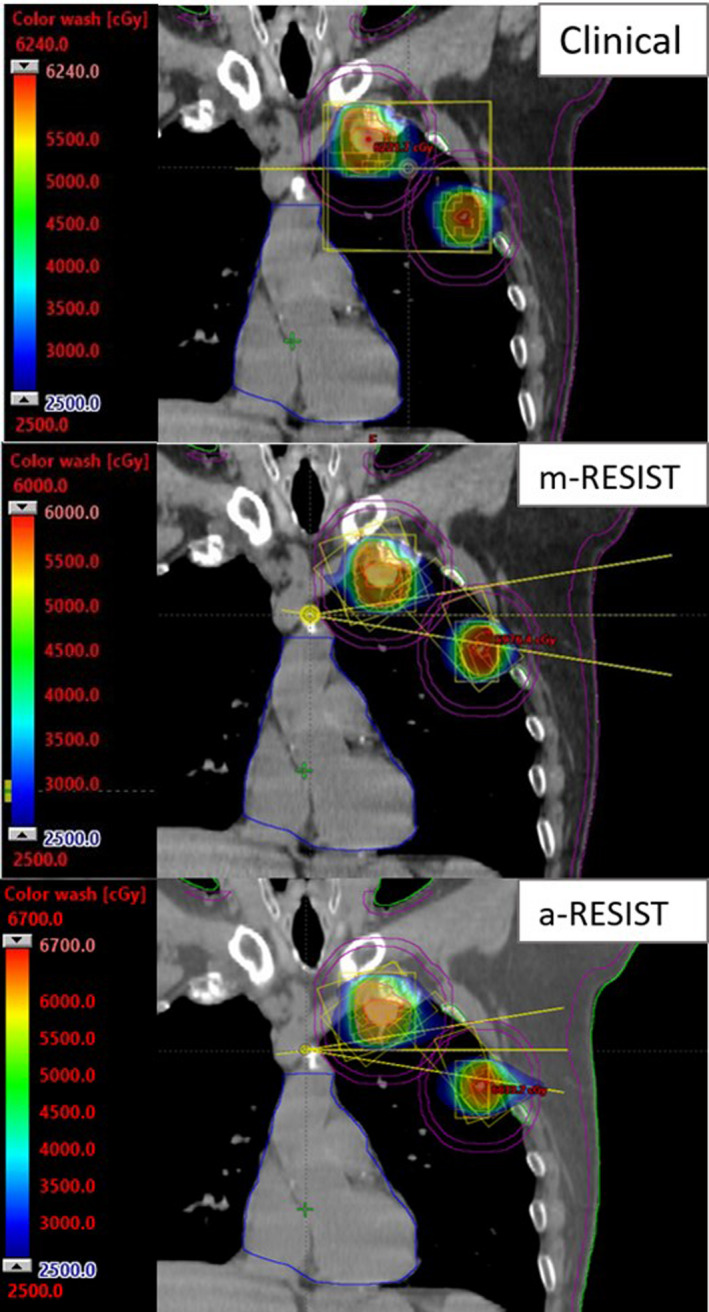
Coronal beam geometry and dose color wash for clinical VMAT, *m*‐RESIST, and *a*‐RESIST plans. Targets shown are PTVs (orange) and GTVs (red). Rings 2 cm away from the PTVs are in purple. OAR shown are skin (purple), heart (blue), ribs (green). The isocenter placement at the patient’s midline for both *m*‐RESIST and *a*‐RESIST allow for non‐coplanar arc geometry, improving planning efficiency and plan quality, escalating tumor dose and avoiding potential collisions.

**Fig. 4:**
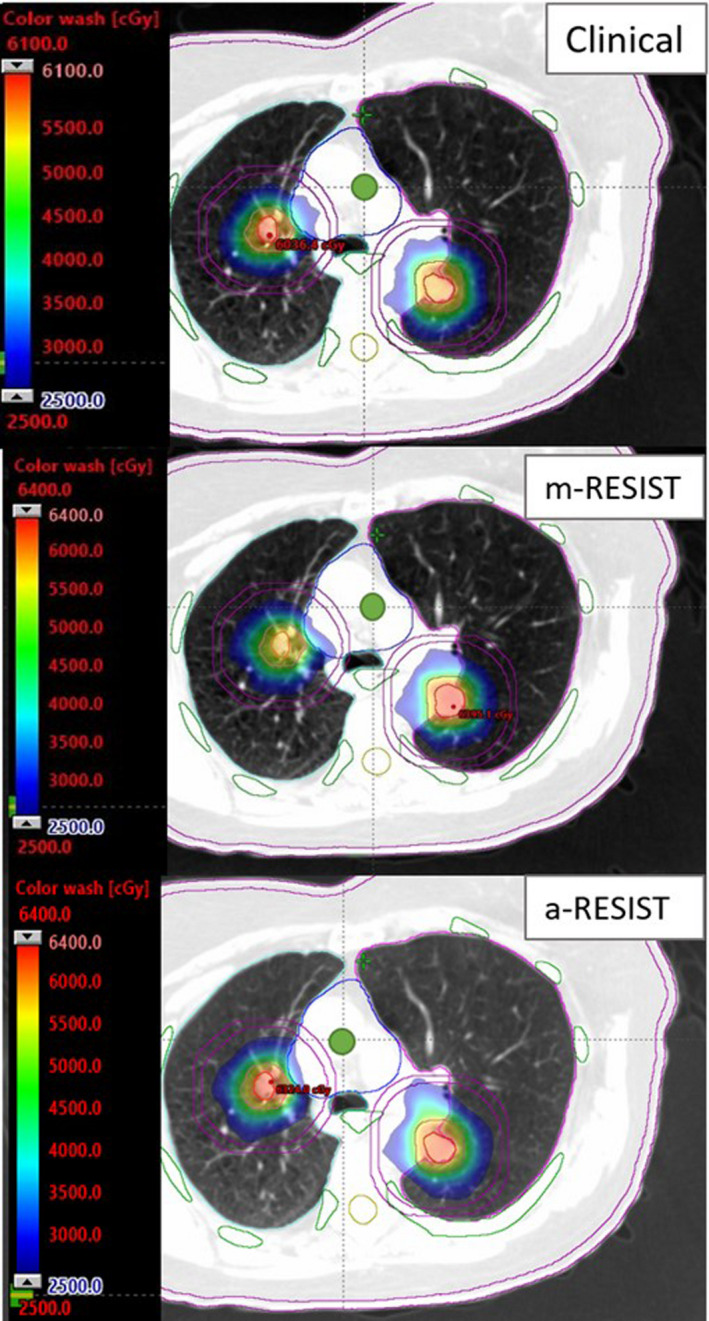
Dose colorwash in the axial plane of a patient’s plan with bi‐lateral lesions near principal bronchial tree. Shown are PTVs (orange), GTVs (red), D2cm ring (purple), ribs (green), heart (blue), esophagus (green), cord (yellow), right lung (blue), left lung (pink), and skin (purple). Green dot at the viewing plane intersection is the isocenter location. The *a*‐RESIST plan provided higher GTV dose and slightly higher intermediate dose spills, which can be seen for the posterior lesion, but it was within the protocol requirements.

The average difference between clinical VMAT and *m*‐RESIST for normal lung V20 Gy, V10 Gy, and MLD was 0.8 ± 0.9% (0.03–2.9%), 3.1 ± 4.3% (−2.0–13.9%), 0.5 ± 0.6 Gy (0.01–1.8 Gy) respectively. Corresponding average difference for those variables between the clinical VMAT and *a*‐RESIST were 0.8 ± 1.9% (−1.6–3.8%), 3.2 ± 7.0% (−2.6–16.4%), 0.3 ± 1.3 Gy (−1.9–2.2 Gy) respectively. These data indicates that, in general, both *m*‐RESIST and *a*‐RESIST can provide better normal lung sparing compared to original clinical VMAT plans. However, occasionally *a*‐RESIST produces plans with slightly higher lung dose as can be seen by the negative values for V20 Gy, V10 Gy, and MLD. This can be attributed to less than ideal isocenter placement and slightly more intermediate dose‐spillage associated with the KBP model used for VMAT optimization. An example of isocenter placement and beam geometry used by all three planning approaches can be seen in Fig. 3 (unilateral lung lesions). For the clinical VMAT plan, the isocenter placement is between the lesions and was planned with co‐planner geometry. The yellow box around the lesions represents the jaw size to cover both lesions. This larger field size is due to treating both lesions at the same time, and although the jaw tracking was enabled, the jaws must track both lesions at once to allow to MLCs travel between the lesions. For both *a‐*RESIST and *m*‐RESIST, the jaw tracking can be utilized more effectively, which can be seen by the small jaw sizes (i.e., jaw tracking locally around each target, one at a time). The upper limit for the dose color wash is much higher for the *a*‐RESIST plan at 67 Gy compared to 60 Gy for *m*‐RESIST and 62.4 Gy for clinical VMAT. This is due to the higher GTV maximum dose obtained with *a*‐RESIST planning approach. The dose color wash in the axial plane of a different patient is demonstrated in Fig. 4 (bilateral lung lesions). For this patient, comparable dose distribution can be seen for all three plans. However, both *m‐*RESIST and *a*‐RESIST exhibited higher dose to GTVs. For the *a‐*RESIST plan, higher intermediate dose spillage can be seen for the posterior lesion.

### 3.B Treatment planning parameters

The average total treatment planning time for the *a‐*RESIST script to complete all ten lung SBRT patients with two lesions was 12.5 ± 3.5 min (9.1–21.1 min). Time was recorded on average of 66 min for *m‐*RESIST plans to complete the same tasks as *a‐*RESIST. The significant reduction of treatment planning time can be attributed to both the automation of arc geometry for *a‐*RESIST as well as the use of the in‐house KBP model for the VMAT optimization. Specifically, the KBP helped create a clinically acceptable and similar plan much quicker than manually inputting and adjusting optimization objectives. As can be seen with slightly higher dose to some OAR and inferior CI, PCN, HI, and D2 cm, *a‐*RESIST plans are less desirable, although clinically acceptable and protocol compliant, compared to *m‐*RESIST. However, the dramatic treatment planning time savings and plan consistency is desirable for a busy clinic. Like *m‐*RESIST plans, *a‐*RESIST plans allow for additional manual intervention to help improve OAR sparing and target coverage with minimal additional treatment planning time, if desired.

### 3.C Treatment delivery parameters

The total number of monitor units for *m‐*RESIST and *a‐*RESIST is about 1.8 times higher than for the clinical VMAT plans, as can be seen in Table 3. However, due to both PTVs being planned separately with separate prescriptions, the average modulation factor for the RESIST methods are lower compared to the clinical VMAT method, could potentially improve treatment delivery accuracy. The estimated treatment time was calculated by adding 10 min for initial patient setup, 1 min to complete a single CBCT (two CBCT scans available with RESIST methods) and about 3 min for tumor‐to‐tumor matching and applying shifts per CBCT. Although the treatment time is longer for both *m‐*RESIST and *a‐*RESIST, these treatments can still be delivered during the typical 30‐min SBRT treatment slot and avoiding the risk of geometric miss.

**Table 3:** Comparison of average values of treatment delivery parameters (and range) between clinical VMAT, *m‐*RESIST, and *a*‐RESIST plans for all ten lung SBRT patients. Mean ± SD (range) was reported. SD = standard deviation.

Beam delivery parameters	Clinical VMAT	*m‐*RESIST	*a*‐RESIST
Total MU per fraction	4020 ± 612 (3091–5010)	7272 ± 1136 (5605–10010)	7065 ± 605 (6021–7982)
Modulation factor (MF)	4.0 ± 0.6 (3.1–5.0)	3.4 ± 0.7 (1.9–5.1)	3.5 ± 0.5 (2.7–4.9)
Beam‐on time (min)	2.8 ± 0.4 (2.2–3.6)	5.2 ± 0.8 (4.0–7.2)	5.0 ± 0.4 (4.3–5.7)
Treatment time (min)	16.8 ± 0.4 (16.2–17.6)	23.2 ± 0.8 (22.0–25.2)	23.0 ± 0.4 (22.3–23.7)

### 3.D Independent pretreatment dose verification

**Fig. 5:**
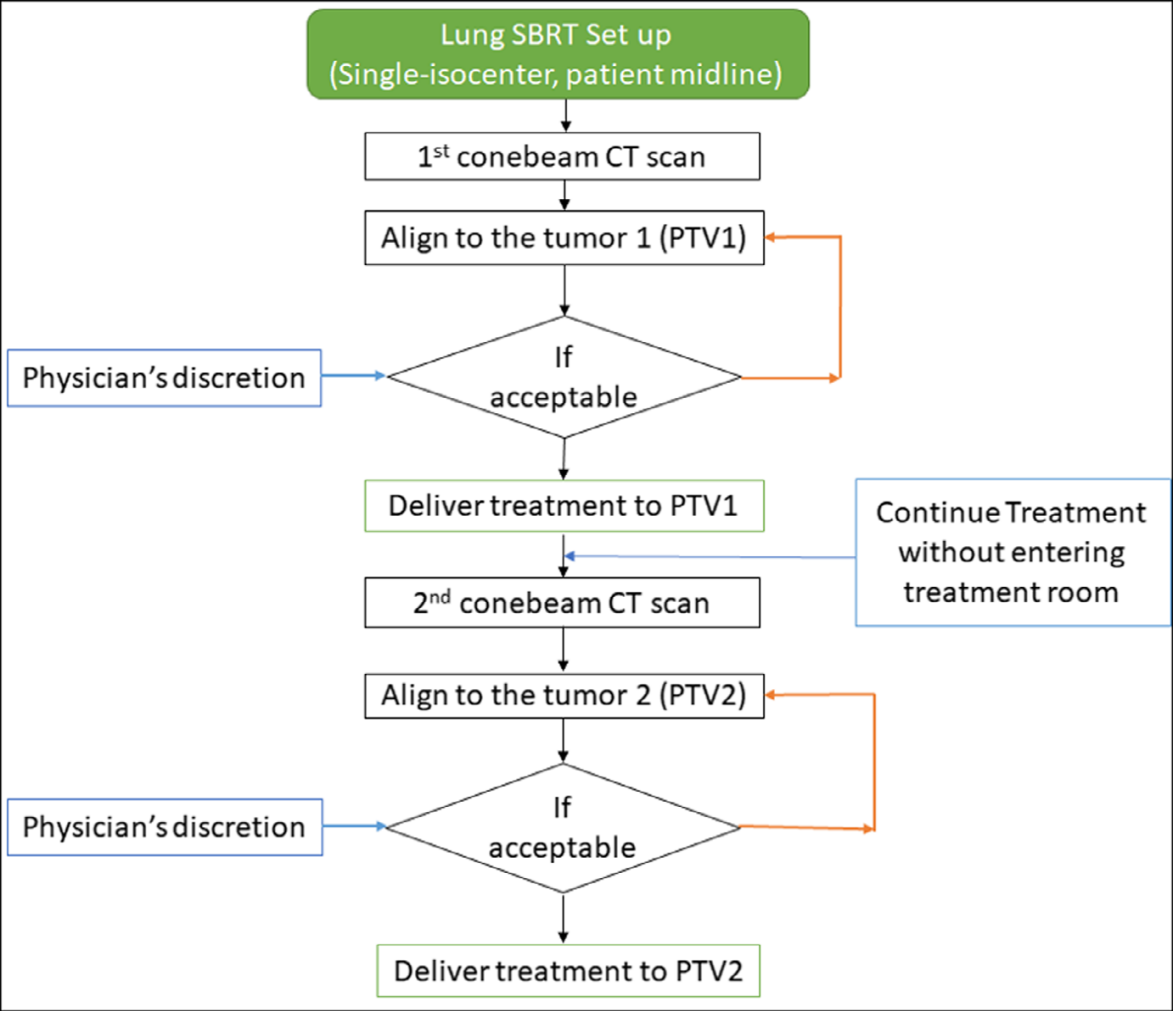
Demonstrated is the *a*‐RESIST treatment delivery workflow for a single‐isocenter/two‐lesion VMAT lung SBRT. The physician has the opportunity to match one lesion at a time and treat without entering the room to re‐setup the patient for the second CBCT thus improving treatment delivery efficiency and accuracy by reducing the chance of a geometric miss. Placement of an isocenter at the mediastinum avoids potential patient/gantry collisions, provides greater flexibility of non‐coplanar arc geometry, and eliminates the need for multiple couch movements during CBCT imaging.

Based on these encouraging results, we have clinically implemented *a*‐RESIST for SBRT treatment of two synchronous lung lesions in our TrueBeam Linac. So far, we have treated four selected lung SBRT patients with two synchronous lung lesions using *a*‐RESIST planning and delivery approach. For these lung SBRT patients, pretreatment dose delivery accuracy was accessed by delivering *a*‐RESIST plans at TrueBeam Linac in the quality assurance (QA) mode using the Octavius QA device that was previously implemented in our clinic for patient‐specific QA procedure. Upon completion of delivered dose, QA datasets were analyzed with Octavius MEPHYSTO Navigator (VeriSoft Patient Plan Verification, Version 6.3, PTW) using the standard clinical gamma passing rate criteria of 2%/2mm maximum dose difference and distance‐to‐agreement with 10% threshold. The dose delivery accuracy of these clinical *a*‐RESIST plans was 96.3 ± 1.7% and 97.1 ± 1.0%, on average for both PTV1 and PTV2, respectively, with 2%/2mm global gamma passing rate criteria ‐ suggesting accurate treatment delivery of the clinical *a*‐RESIST plans. For all a‐RESIST patients, saily pretreatment CBCT images were acquired for each lesion and soft tissue matching of tumor‐to‐tumor one at a time (see Fig. 5). Pretreatment CBCT imaging couch corrections of four selected a‐RESIST lung SBRT patients with two synchronous lesions were less than ±2 mm and ±2° in each direction for each lesion. Those corrections were applied to each lesion, one at a time and approved by the treating physicians before delivering the SBRT treatments.

## 4 DISCUSSION

This report describes and assesses the feasibility of the automated RESIST method for treating two synchronous lung lesions with SBRT that aims to minimize setup uncertainties and significantly improve treatment planning time. First, the method was validated by retroactively planning ten patients with two lesions who previously underwent VMAT SBRT with a single‐isocenter placed between the two lesions. Secondly, after the phantom’s tests and further validation, we have clinically implemented a‐RESIST for synchronous two lesions lung SBRT patients. RESIST consists of a single‐isocenter placed at the patient’s midline and both lesions sharing the same isocenter. Unlike clinical VMAT, in RESIST plans both lesions have an individual plan which are then evaluated with a plan summation. Allowing each lesion to be planned individually allows for optimal collimator angles and the best use of the jaw tracking feature to aid in the reduction of the normal lung dose. Furthermore, two plans sharing the same isocenter allows for more flexibility during patient treatment as demonstrated in Fig. 5 and therapists does not need to enter the treatment room for resetting the patient for the second tumor. Utilizing *a*‐RESIST could potentially decreases the chance of a geometric target miss by allowing daily pretreatment CBCT soft tissue matching of one tumor at a time. Moreover, we believe that this way, the spatial setup error of about 1 mm at 10 cm distance from the isocenter could be easily accommodated by the generous PTV margin to each lesion of 5 to 10 mm. Additionally, utilizing 3‐partial arcs geometry (at 0°, ±10° couch positions), the interplay effect of a change in breathing patterns with MLC modulation, gantry rotation, and dose‐rate changes during dose delivery that could likely average out, as demonstrated by Ong et al.^21^ that the interplay effect causes insignificant dose blurring when using more than two arcs.

Single‐isocenter VMAT plans have become a popular treatment option for intracranial stereotactic radiosurgery (SRS) and more recently, are becoming of interest for extracranial lesions. A few studies have demonstrated the use and feasibility of treating multiple lung lesions with a single isocenter approach.^22^, ^23^ However, these studies fail to acknowledge the treatment planning difficulties, neither provided solutions to treatment delivery uncertainties, nor provided a clear guidance to the therapists for better clinic work flow. The *a*‐RESIST planning method decreases the treatment planning difficulties for two lesions lung SBRT plans as well as provides appropriate guidance for more accurate treatment delivery, eliminating unwanted stress on the entire SBRT team. Furthermore, to our knowledge, *a*‐RESIST is the first approach to automate single‐isocenter/multiple‐lesion lung SBRT plans. Automated treatment planning is a fast‐developing area of research and, with the recent availability of writeable scripting using Varian ESAPI, will continue to gain favor. A recent study demonstrated the use of automation in an existing clinical workflow and showed the feasibility of automation for improving clinical efficiency and safety for total body irradiation (TBI).^24^ Similar to TBI procedures, lung SBRT procedures are high risk, involving large doses per fraction. The *a*‐RESIST method can be used to reduce treatment planning errors and potentially reduce the chance of tumor misalignment for treatment by aligning soft‐tissues tumor matching one at a time. Recent publications have discussed the challenges of lung SBRT plan optimization including multicenter plan comparison^25^, ^26^ and explored automation of treatment planning for various treatment of other sites^27^, ^28^, ^29^ although *a*‐RESIST is the first of its kind for multiple lesions VMAT lung SBRT treatment. Based on these results, we have further validated *a*‐RESIST via pretreatment QA measurement on our selected four new patients with two synchronous lesions and implemented clinically for lung SBRT treatments. However, *a*‐RESIST still needs a pretreatment “dry‐run” at the machine before patient’s treatment to make sure that there is no collision issue with the patient’s plan. Treatment outcome and clinical follow‐up results of multilesions patients treated via *a*‐RESIST method for larger patient cohort is anticipated.

Further improvement of *a*‐RESIST is ongoing in our center including improvement of the KBP optimization model for two‐lesion lung SBRT plans and standardizing a more “patient‐specific” approach to isocenter placement that could minimize tumor distance to isocenter, while still keeping the patient’s midline isocenter. Simulated collision detection is a feature available when using Varian HyperArc module for intracranial SRS treatments and has been further developed by the multiple researchers.^30^, ^31^, ^32^ However, simulated collision detection for extracranial SBRT has yet to be studied and would be the next step to the *a*‐RESIST method to further ensure an efficient treatment delivery by automatic collision detection, further reducing overall treatment time. Efficacy of *a*‐RESIST has been demonstrated for two synchronous lung lesions SBRT that could potentially allow for offline adaptive replanning (if required) and can potentially be used for more than two lung lesions as well as other extracranial treatment sites, such as multilesion liver SBRT or oligometastastic abdominal/pelvic lymph nodes SBRT.

## 5 CONCLUSION

Using the *a*‐RESIST planning method for synchronous lung lesions can significantly decrease treatment planning time (<20 min) and allow planners to create clinically acceptable lung SBRT plans. The RESIST method reduces the chance of a geometric miss due to setup uncertainties by allowing for planning and setup of each lesion individually, permitting tumor‐to‐tumor matching on daily CBCT. Furthermore, automation of the planning technique will allow for standardized treatment plans while allowing user input to further increase the plan quality and treatment efficiency. Utilizing an in‐house trained lung SBRT RP model helps ensure that treatment plans are of consistent high quality. Further improvement of the *a‐*RESIST script to ensure more precise patient specific isocenter placement algorithm as well as well‐trained KBP models for patient‐specific multitargets lung SBRT that could further improve plan quality, reduce inter‐planner variability and inconsistency, and improve patient safety and clinic workflow–potentially allowing for offline adaptive replanning is desired.

## CONFLICT OF INTEREST

The authors declare no conflict of interest.

## FUNDING

None.

## AUTHOR’S CONTRIBUTION

DP and LCC designed the project. LCC wrote the *a*‐RESIST script, collected and analyzed the data. DP, RM, MR, and MB provided clinical expertise and supervision of the paper. JV and DP developed the lung SBRT RapidPlan model. LCC and DP drafted the manuscript and all co‐authors revised and approved the final manuscript.

## Data Availability

Research data are not shared.
